# Supplementary material for: Fabrication and Characterization of a Flexible Non-Enzymatic Electrochemical Glucose Sensor Using a Cu Nanoparticle/Laser-Induced Graphene Fiber/Porous Laser-Induced Graphene Network Electrode
Source: Sensors (Basel). 2025 Apr 7;25(7):2341. doi: 10.3390/s25072341 (PMC11991655; doi:10.3390/s25072341)
Supplement: Supplementary file 1 [file sensors-25-02341-s001.zip › sensors-3503703-supplementary.pdf]

Supplementary Materials of the following article:

Fabrication and Characterization of a Flexible Non-Enzymatic Electrochemical Glucose Sensor Using a Cu Nanoparticle/Laser-Induced Graphene Fiber/Porous Laser-Induced Graphene Network Electrode

Taeheon Kim and James Jungho Pak *

School of Electrical Engineering, Korea University, Seoul 136-713, Republic of Korea; page21c@korea.ac.kr
* Correspondence: pak@korea.ac.kr; Tel.: +82-2-3290-3238; Fax: +82-2-921-0544

Keywords: laser-induced graphene; flexible sensor; Cu nanoparticle; non-enzymatic electrochemical detection

Table S1. Comparison of carbon, nitrogen, and oxygen atomic percentages of LIGF/LIG electrodes fabricated at laser powers of 8.5 W, 8.6 W, 8.7 W, 8.8 W, and 8.9 W.

	**Carbon (C)**		**Nitrogen (N)**		**Oxygen (O)**	
Laser Power (W)	Intensity	Atomic Percentage (%)	Intensity	Atomic Percentage (%)	Intensity	Atomic Percentage (%)
8.5	36627	93.7	967.5	0.94	3517	5.4
8.6	35771	94.7	618.9	0.62	2948.3	4.6
8.7	36989	95.4	1103.1	1.1	2313	3.5
8.8	39251	95.7	770.5	0.71	2469.1	3.5
8.9	42500	98.5	287.2	0.25	936.3	1.3
9.0	35500	94.7	772	0.78	2830.5	4.5

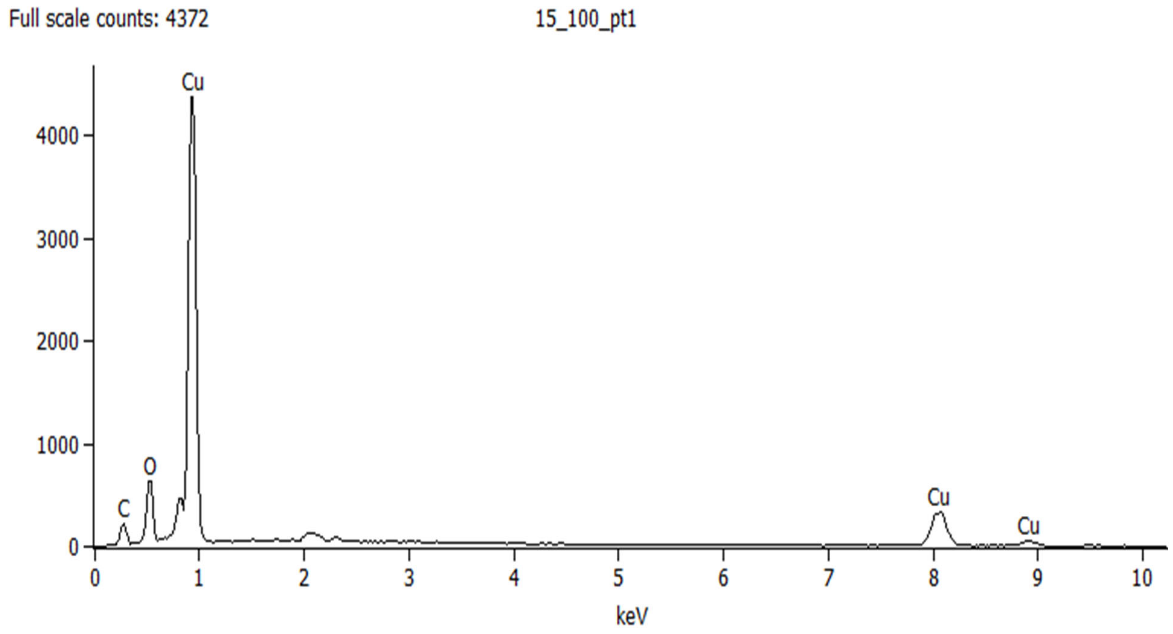

Figure S1. EDS spectra of the Cu NP-coated LIGF/LIG electrode.

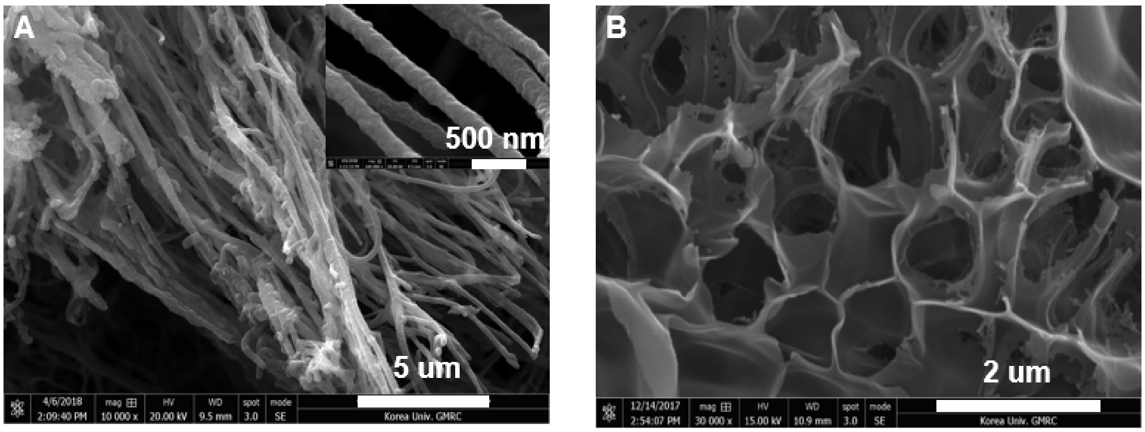

Figure S2. FE-SEM images of (A) a Cu-NP-coated LIGF (×10k) and (B) a porous LIG electrode (×20k).

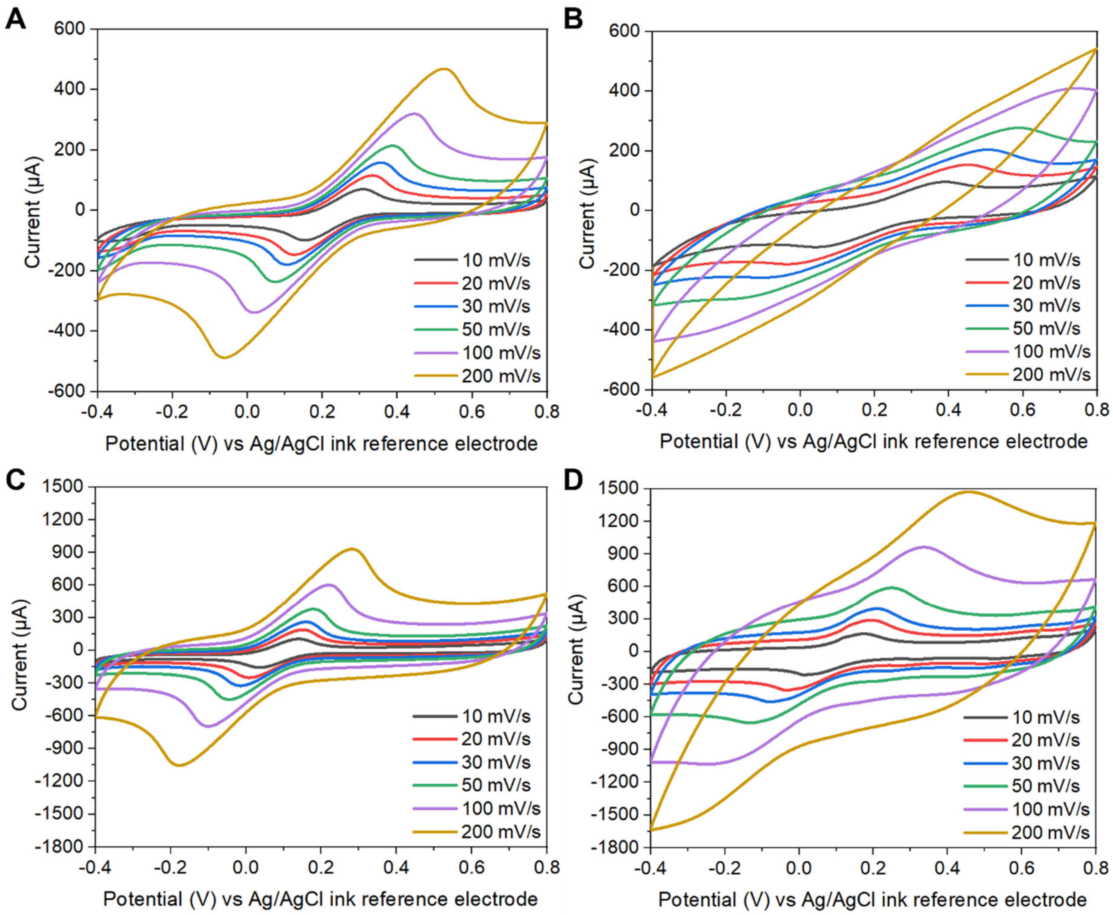

Figure S3. CV experiments using (A) bare LIG, (B) Cu NP/LIG, (C) bare LIGF/LIG, and (D) Cu NP/LIGF/LIG electrodes were conducted in [10 mM $K_3[Fe(CN)_6]$ + 1 M KNO_3] (1:1) ferricyanide solution at different scan rates from -0.4 to + 0.8 V to investigate the electrochemical reaction.

Table S2. Comparison of the glucose sensor oxidation peak current change rate with respect to the peak current at under different bending angles: 0°, 45°, 90°, 135°, and 180°.

Glucose Concentration (mM)	Bending Angle (°)	Average Current (uA)	Oxidation Peak Current Change Rate (%)
1	0	596.0	reference value
	45	573.6	- 3.7
	90	551.0	- 7.5
	135	527.6	- 11.4
	180	508.3	- 14.7
2	0	711.3	reference value
	45	680.6	- 4.3
	90	657.6	- 7.5
	135	638.0	- 10.3
	180	607.3	- 14.5
3	0	825.0	reference value
	45	787.3	- 4.5
	90	753.5	- 8.6
	135	721.4	- 12.5
	180	684.0	- 17.1
4	0	993.6	reference value
	45	954.6	- 3.9
	90	913.0	- 8.1
	135	864.1	- 13.0
	180	817.0	- 17.7
